# Low-Dose Oral Sirolimus and the Risk of Menstrual-Cycle Disturbances and Ovarian Cysts: Analysis of the Randomized Controlled SUISSE ADPKD Trial

**DOI:** 10.1371/journal.pone.0045868

**Published:** 2012-10-10

**Authors:** Matthias Braun, James Young, Cäcilia S. Reiner, Diane Poster, Fabienne Krauer, Andreas D. Kistler, Paulus Kristanto, Xueqi Wang, Yang Liu, Johannes Loffing, Gustav Andreisek, Arnold von Eckardstein, Oliver Senn, Rudolf P. Wüthrich, Andreas L. Serra

**Affiliations:** 1 Division of Nephrology, University Hospital, Zürich, Switzerland; 2 Biometrical Practice BIOP AG, Basel, Switzerland; 3 Division of Diagnostic and Interventional Radiology, University Hospital, Zürich, Switzerland; 4 Medication Adherence Research Centre, AARDEX Group, Visé, Belgium; 5 Department of Nephrology, Changzheng Hospital, Shanghai, China; 6 Center for Integrative Human Research, University of Zürich, Zürich, Switzerland; 7 Institute of Anatomy, University of Zürich, Zürich, Switzerland; 8 Institute of Clinical Chemistry, University Hospital, Zürich, Switzerland; 9 Institute of General Practice and Health Services Research, University Hospital, Zürich, Switzerland; Omaha Veterans Affairs Medical Center, United States of America

## Abstract

Sirolimus has been approved for clinical use in non proliferative and proliferative disorders. It inhibits the mammalian target of rapamycin (mTOR) signaling pathway which is also known to regulate ovarian morphology and function. Preliminary observational data suggest the potential for ovarian toxicity but this issue has not been studied in randomized controlled trials. We reviewed the self-reported occurrence of menstrual cycle disturbances and the appearance of ovarian cysts *post hoc* in an open label randomized controlled phase II trial conducted at the University Hospital Zürich between March 2006 and March 2010. Adult females with autosomal dominant polycystic kidney disease, an inherited kidney disease not known to affect ovarian morphology and function, were treated with 1.3 to 1.5 mg sirolimus per day for a median of 19 months (N = 21) or standard care (N = 18). Sirolimus increased the risk of both oligoamenorrhea (hazard ratio [HR] 4.3, 95% confidence interval [CI] 1.1 to 29) and ovarian cysts (HR 4.4, CI 1.1 to 26); one patient was cystectomized five months after starting treatment with sirolimus. We also studied mechanisms of sirolimus-associated ovarian toxicity in rats. Sirolimus amplified signaling in rat ovarian follicles through the pro-proliferative phosphatidylinositol 3-kinase pathway. Low dose oral sirolimus increases the risk of menstrual cycle disturbances and ovarian cysts and monitoring of sirolimus-associated ovarian toxicity is warranted and might guide clinical practice with mammalian target of rapamycin inhibitors.

**Trial Registration:**

ClinicalTrials.gov NCT00346918

## Introduction

Sirolimus (Rapamune, Pfizer, New York, NY, USA) is a potent immunosuppressive and anti-proliferative drug which blocks the mammalian target of rapamycin (mTOR). MTOR is a key regulatory kinase which is also known to regulate ovarian function [1]. The drug has been approved by both the US Food and Drug Administration and the European Medicines Agency for the prevention of renal allograft rejection.

Most of our knowledge regarding sirolimus toxicity has been derived from kidney transplant efficacy trials. However there were no reports of menstrual cycle disturbances and ovarian cyst formation in three large clinical trials [2], [3], [4], although these adverse events were reported in three case series [5], [6], [7]. Ovarian dysfunction is difficult to recognize in clinical trials: symptoms are often non-specific and can be wrongly attributed to concomitant medication or comorbidities [8].

Aside from organ transplantation, sirolimus is being assessed for clinical effectiveness in several cancers and in other proliferative disorders, including autosomal dominant polycystic kidney disease (ADPKD). ADPKD is characterized by the growth of kidney cysts; the disease itself is not known to affect ovarian morphology and function [9], [10], [11]. Although sirolimus shows promise in rodent polycystic kidney disease models [12], [13], [14], [15], [16], [17], 18 months treatment with sirolimus did not slow the growth of kidney cysts in adults with ADPKD [18].

Animal and observational data suggest the potential for ovarian toxicity but this issue has not been considered in previous trials [19]. We sought to determine whether sirolimus causes menstrual cycle disturbances and ovarian cysts in adults with ADPKD enrolled in a randomized controlled trial. Here we extend a letter [20] warning of an increased risk of ovarian toxicity among patients receiving sirolimus; we provide full details and suggest a possible mechanism of toxicity.

## Methods

### Trial Setting

The SUISSE ADPKD study was a randomized controlled phase II trial carried out to determine whether 18 months of treatment with sirolimus slows kidney growth in adults with ADPKD. The occurrence of menstrual cycle disturbances and ovarian cysts were pre-defined secondary endpoints in the statistical analysis plan [18]. From March 2006 through March 2008 we enrolled 100 patients (39 females) with ADPKD at the University Hospital Zurich [21]. These patients were between 18 and 40 years of age, with an estimated creatinine clearance of at least 70 milliliter per minute. The trial was run according to the principles of the Declaration of Helsinki, the Good Clinical Practice guidelines of the International Conference on Harmonization, and local regulatory requirements. The protocol for this trial and supporting CONSORT checklist are available as supporting information; see Checklist S1 and Protocol S1. The medical ethics committee of the Canton Zürich, Switzerland (SPUK) approved the trial protocol [22]. All patients gave written informed consent.

After a run-in period of 6 months, patients were randomly assigned to receive either 18 months treatment with sirolimus (target dose 2 mg daily) or standard care. Standard care consisted of blood pressure control (office systolic and diastolic blood pressure targets below 130 and 85 mm Hg respectively), prompt antibiotic treatment of kidney cyst infections, and avoidance of potentially nephrotoxic substances. The sirolimus dose was adjusted to achieve steady-state levels between 4 and 10 µg per liter, determined by liquid chromatography–mass spectrometry from whole blood. Patient adherence to sirolimus was assessed using an electronic system (MEMS™, Aardex Group, Ltd., Sion, Switzerland).

Wyeth (now Pfizer) had no role in the trial design, collection, analysis and interpretation of the data or the writing of the report. Andreas L. Serra and James Young had full access to the study data and take responsibility for the integrity of the data and the accuracy of the data analysis.

### Assessment of trial outcomes

At enrollment, randomization, 6, 12 and 18 months patients were asked if they had any menstrual cycle abnormalities in the past 6 months. Oligoamenorrhea was defined as no menstrual period for 3 months or more when one should have occurred, or an interval of more than 35 days between menstrual periods. Our definition of oligoamenorrhea was based on criteria used in epidemiologic studies [23], [24].

Abdominal magnetic resonance imaging (MRI) without contrast material was performed every 6 months using a 1.5T MR scanner (Signa Echospeed Excite HD or HDx, General Electrics (GE) Healthcare, Waukesha, Wisconsin, USA).[25] At each point in time, the number and diameter of right and left ovarian cysts greater than 2 cm in diameter were measured on coronal T2-weighted single-shot fast spin echo sequences acquired in breath hold technique using a dedicated computer workstation (Advantage Windows Workstation 4.4, GE Healthcare). The workstation observer was blinded to all clinical data and patients were measured in random order.

### Statistical Analysis

Analyses of prevalence and incidence were carried out using logistic and Cox proportional hazards regression in SAS version 9.2 (Cary, NC, USA). Outcomes for logistic regression were any patient report of oligoamenorrhea and any detected ovarian cyst greater than 2 cm in diameter during the 18 months treatment period. Outcomes for Cox proportional hazards regression were time to a first report of oligoamenorrhea in those without oligoamenorrhea at randomization, and time to a first ovarian cyst greater than 2 cm in diameter in those without such cysts at randomization. Since these outcomes can only be assessed at each visit we used a discrete time version of the Cox proportional hazards model.[26] For both logistic and proportional hazard regression, we report profile likelihood and exact confidence intervals. With few events, maximum likelihood estimates for odds and hazard ratios tend to be biased away from a value of one [27], [28] however, exact confidence intervals tend to be wider than is strictly necessary [29], [30]. With few events, it is not sensible to include covariates in any of our models and we estimate only the effect of the randomized treatment on outcome. The discrete time version of the Cox proportional hazards model has a complementary log-log link function and an offset to adjust for some slight variation in the time between visits scheduled 6 months apart [26]. However the exact estimate must be based on a model with a logit link function and no offset.

### Animal Studies

The sirolimus-associated effects on the mTOR signaling pathway in ovaries were examined by Western blotting and immunohistochemistry in rats given oral sirolimus. The animal study was approved by the animal health regulatory agency of the Canton Zürich, Switzerland.

### Animal Study Procedures

Female 4 week old Wistar rats were given daily 3.0 milligram per kilogram body weight sirolimus (N = 4) or vehicle (N = 4) by gavage feeding. The rats were sacrificed during pro-estrus three weeks after initiation of treatment at six PM and serum levels of follicle and luteal stimulating hormones were measured by enzyme linked immunoassay according to the manufactures' protocol.

### Western Blots

Ovaries were homogenized with ice-cold lysis buffer containing 40 mM Hepes, 120 mM NaCl, 1 mM ethylenediaminetetraacetic acid (EDTA), 10 mM potassium pyrophosphate, 10 mM glycerol phosphate, 50 mM NaF, 0.5 mM NaVO3, 1% Triton, and protease inhibitor (pH 7.6). Tissue lysates were cleared by centrifugation. Equal amounts of lysates were resolved on sodium dodecyl sulfate polyacrylamide gel electrophoresis (SDS-PAGE), transferred to nitrocellulose membranes, and probed with antibodies. Cell Signaling Technology supplied antibodies against Akt ^Ser473^, p70 S6K ^Thr421/Ser424^ and p70 S6K; Abcam supplied an anti-Akt antibody; Millipore supplied an anti- glyceraldehyde-3-phosphate dehydrogenase (GAPDH) antibody.

### Immunohistochemistry

Paraffin-embedded sections were cut and stained by the immunoperoxidase technique, following standard methods of deparaffinization, antigen retrieval, primary antibody incubation with either anti-Akt Ser473 (Cell Signaling) or anti-p70 S6K Thr421 (GenWay), secondary antibody incubation, 3,3-diaminobenzidine (DAB) incubation, and counterstaining with methyl green.

## Results

### Patients

**Figure 1:**
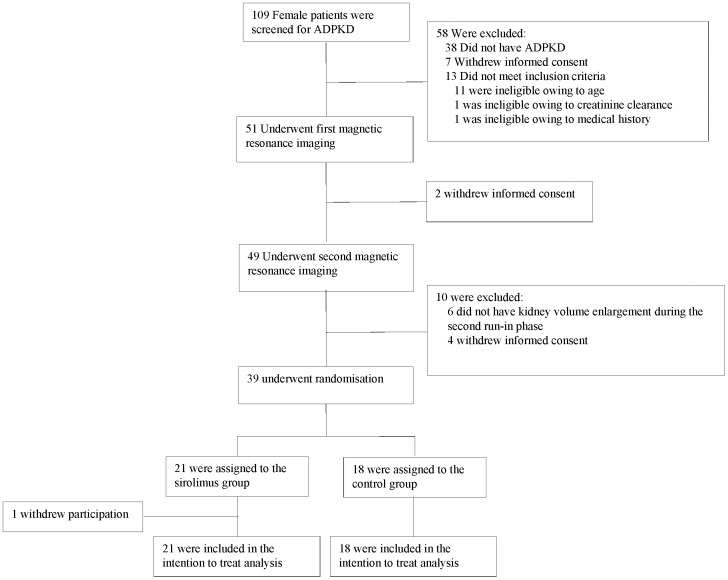
Enrollment and Outcomes.

Of the 39 females enrolled, 21 were randomized to receive sirolimus and 18 to receive standard care. Follow-up was complete in all but one patient receiving sirolimus who withdrew at 6 months (**Figure 1**). At randomization, patient characteristics were similar between the groups (**Table 1**): 2 patients in the sirolimus group and 1 patient in the control group reported at least one episode of oligoamenorrhea in the last 6 months (**Table 2**). At randomization, ovarian cysts were recorded in 2 patients in the sirolimus group and in 4 patients in the control group (**Table 2**).

**Table 1:** Characteristics of patients at randomization.*

Characteristic – values are means (standard deviation) or numbers (percent)	Sirolimus	Control
	(N = 21)	(N = 18)
**Age** – year	31 (8)	32 (7)
**BMI** – kilogram per m^2^ †	22 (3)	22 (3)
**Blood pressure** – mm Hg		
Systolic	132 (12)	120 (11)
Diastolic	83 (11)	78 (10)
**Estimated GFR** – ml/min per 1.73 m^2^ §	88 (20)	96 (16)
**Chronic kidney disease stage** – no. (%)¶		
1	10 (48)	11 (61)
2	10 (48)	7 (39)
3	1 (5)	0
**Menarche** – year‡	13 (2)	13 (2)
**Parity** – no. (%)‡		
Nulliparous	13 (62)	14 (78)
1–2	7 (33)	4 (22)
>2	1 (5)	0
**Contraception** – no. (%)‡		
Hormonal	10 (48)	9 (50)
Barrier method	11 (52)	9 (50)

* From New England Journal of Medicine, Ovarian toxicity from sirolimus, Braun M, Young J, Reiner CS, Poster D, Wüthrich RP, Serra AL. 366(11):1062-4. Copyright © (2012) Massachusetts Medical Society. Reprinted with permission.

† The body-mass index (BMI) is the weight in kilograms divided by the square of the height in meters.

§ The glomerular filtration rate (GFR) was estimated by using the Chronic Kidney Disease Epidemiology Collaboration (CKD-EPI) equation [48].

¶ Chronic kidney disease was classified according to the Kidney Disease Outcomes Quality Initiative of the National Kidney Foundation. Stage 1 denotes a glomerular filtration rate (GFR) of 90 ml or more per minute per 1.73 m^2^ of body-surface area; stage 2, a GFR of 60 to 89 ml per minute; and stage 3, a GFR of 30 to 59 ml per minute.

‡ Patients' self-reported medical histories at randomization.

**Table 2:** Number of patients with oligoamenorrhea and ovarian cysts.*

Number of patients (percent) [missing if any]	Sirolimus N = 21	Control N = 18
**Oligoamenorrhea** ‡		
Enrollment	3 (14)	2 (12) [1]
Randomization	2 (10) [1]	1 (7) [3]
6 months	9 (43)	2 (11)
12 months	5 (25) [1]	2 (11)
18 months	5 (26) [2]	1 (6)
Any visit after randomization	11 (52)	3 (17)
**Ovarian cysts** †		
Enrollment	4 (19)	4 (25) [2]
Randomization	2 (10)	4 (24) [1]
6 months	6 (29)	2 (14) [4]
12 months	6 (30) [1]	3 (18) [1]
18 months	7 (39) [3]	2 (12) [1]
Any visit after randomization	12 (57)	5 (28)

* From New England Journal of Medicine, Ovarian toxicity from sirolimus, Braun M, Young J, Reiner CS, Poster D, Wüthrich RP, Serra AL. 366(11):1062-4. Copyright © (2012) Massachusetts Medical Society. Reprinted with permission.

‡ Patients' self-reported events during the 6 months before the visit.

† Assessed by two independent observers – there was no disagreement between observers.

Median (interquartile range [IQR]) follow up time after randomization was 19 months (19–20) and 19 months (18–20) in the sirolimus and control groups, respectively. Both the numbers of missing reports of menstrual cycle disturbances and the number of unreadable MRI scans were lower among patients in the sirolimus group (**Table 2**).

### Trial Outcomes

**Figure 2:**
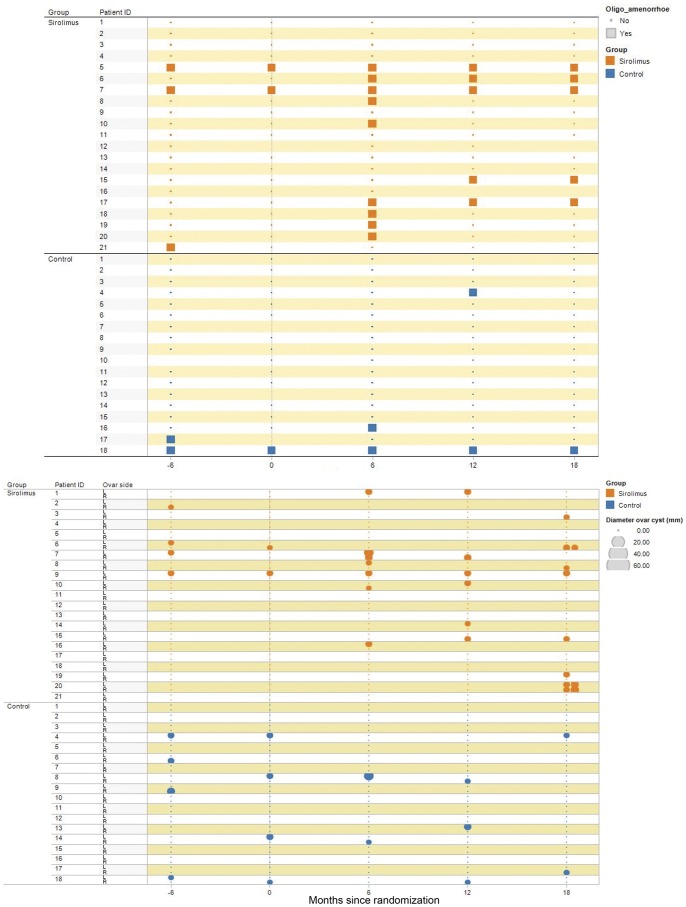
Oligoamenorrhea (upper panel) and the size (lower panel; in millimeters) and the left-right location (R, L) of ovarian cysts over time in the sirolimus (orange) and control (blue) groups.

A total of 11 out of 21 patients in the sirolimus group reported oligoamenorrhea at any visit after randomization, compared to 3 out of 18 patients in the control group (**Table 2**). Five patients in the sirolimus group reported more than one episode of oligoamenorrhea during the trial whereas only one patient in the control group reported more than one episode of oligoamenorrhea (**Figure 2**).

Ovarian cysts were observed in 12 out of 21 patients in the sirolimus group, compared to 5 out of 18 patients in the control group (**Table 2**). Six patients in the sirolimus group had ovarian cysts at more than one visit after randomization compared to only two patients in the control group (**Figure 2**). One patient presented with acute abdominal pain and a large cyst of the left ovary while receiving sirolimus and was cystectomized at 164 days after randomization.

Differences in cycle disturbances were apparent in those not on oral contraceptives – 8 out of 11 and 2 out of 9 patients in the sirolimus and control groups; but were less apparent in those on oral contraceptives – 3 out of 10 and 1 out of 9 patients in the sirolimus and control groups. Differences in ovarian cysts between sirolimus and control did not seem to depend on the contraceptive method (barrier methods: 7 out of 11 and 3 out of 9 patients in the sirolimus and control groups; oral contraceptives: 5 out of 10 and 2 out of 9 patients in the sirolimus and control groups).

Although imprecise, estimates of odds and hazard ratios suggest that the prevalence and incidence of both oligoamenorrhea and ovarian cysts were higher among patients receiving sirolimus (**Table 3**).

**Table 3:** The effect of treatment with sirolimus on the prevalence and incidence of oligoamenorrhea and ovarian cysts: odds and hazard ratios from logistic and Cox proportional hazard regression.

	Oligoamenorrhea		Ovarian cysts	
	Estimate	95% CI	Estimate	95% CI
**Logistic regression – odds ratios**				
Profile likelihood	5.5	1.3 to 29	3.5	0.94 to 14
Exact	5.3	1.0 to 37	3.4	0.76 to 17
**Cox proportional hazards regression – hazard ratios**				
Profile likelihood	4.3	1.1 to 29	4.0	1.1 to 26
Exact*	4.4	0.75 to 48	4.3	0.82 to 43

* Exact hazard ratios were estimated using a model with a logit link function and no offset. Profile likelihood estimates for this alternative model were 4.5, (95% confidence interval [CI] 1.1 to 31) and 4.6, (95% CI 0.99 to 34) for oligoamenorrhea and ovarian cysts respectively.

### Adherence to Sirolimus

**Figure 3:**
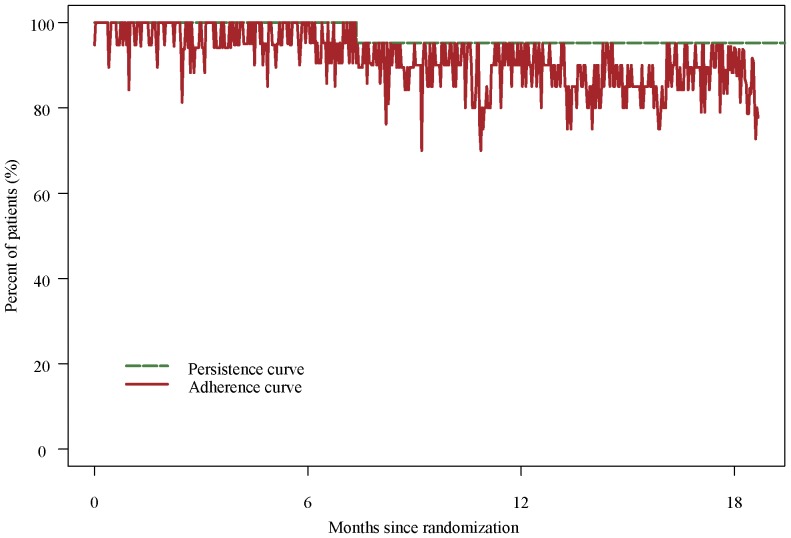
Time course of adherence parameters (persistence and adherence) in patients assigned to the sirolimus group. The persistence curve shows a Kaplan Meier estimation of the proportion of patients on treatment. The adherence curve indicates the day-to-day proportion of patients who took sirolimus as prescribed. Data from 21 individual dosing histories were used for this analysis.

Mean sirolimus doses were between 1.2 and 1.5 milligram per day and the sirolimus steady-state blood levels between 3.5 and 4.7 microgram per liter (**Table 4**). Patient adherence to sirolimus, measured by percentage of correct dosing, was 95% over the 18 month treatment period (**Figure 3**).

**Table 4:** Sirolimus dosage, sirolimus steady-state blood level and parameters of treatment adherence in patients assigned to the sirolimus group.

Months	Sirolimus dosage* (milligram per day)	Sirolimus level* (microgram per liter)	Continuation† (%)	Adherence‡ (%)
**3 months**	1.1 (0.5)	3.5 (1.0)	100	97.6
**6 months**	1.3 (0.4)	3.5 (1.1)	100	96.9
**9 months**	1.2 (0.6)	3.7 (1.1)	95.2	95.1
**12 months**	1.4 (0.4)	4.2 (1.9)	95.2	93.9
**18 months**	1.5 (0.5)	4.7 (1.6)	95.2	92.2

* Values are means (standard deviation).

† Continuation was defined as the proportion of patients who remained on the sirolimus treatment.

‡ Adherence was defined for each specific time interval as the average daily proportion of patients who took their sirolimus dose as prescribed among those still on the sirolimus treatment.

### Animal Studies

**Figure 4:**
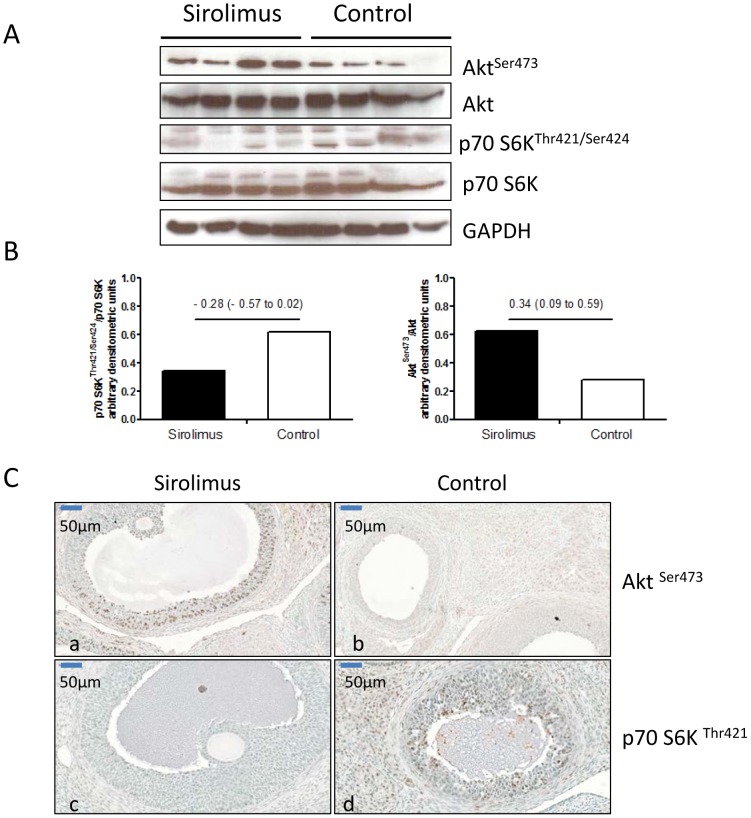
Molecular (Panel A and B) and imunohistochemical analyses (Panel C) of rat ovary. Panel A. Gel electrophoresis of proteins carried out to assess sirolimus-associated changes in the activity of key signaling pathways thought to regulate ovarian function and morphology. Sirolimus treatment amplified signaling through phosphatidylinositol 3-kinase (Akt^Ser743^), thought to be associated with human polycystic ovarian syndrome, and blocked the mammalian target of rapamycin pathway (p70 S6K^Thr421^). Panel B. The ratios of phosphorylated to non-phosphorylated molecules are expressed in arbitrary densitometric units (Sirolimus minus control – mean (95% confidence interval): p70 S6K^Thr421/Ser424^ to p70 S6K −0.28 (−0.57 to 0.02) and Akt^Ser473^ to Akt 0.34 (0.09 to 0.59). Panel C: Immunohistochemical analysis shows positive staining with a nuclear pattern for Akt^Ser743^ in granulosa cells of rats receiving sirolimus and for p70 S6K^Thr421^ in granulosa cells of control rats receiving vehicle.

Rats given sirolimus 3.0 milligram per kilogram per day for three weeks, a dose that produces blood concentrations similar to those in patients [31], had lower body weight: 260±14 g (mean ± standard deviation) in the sirolimus group and 291±17 g in the control group. The ratios of ovaries to body weight were similar between groups: 0.045±0.013 in the sirolimus group and 0.039±0.001 in the control group. We did not find evidence for an increased frequency of ovarian cysts: two observers blinded for the treatment allocation reviewed the histological sections and reported no difference in the number and morphology of ovarian follicles. The automatically calculated ratios of blank space to total ovarian area on histological slides, as a proxy for ovarian cysts, were similar in both groups: 9.5±2.7% in the sirolimus group and 8.5±2.4% in the control group. The cycle lengths were similar both groups: 7±4 days in the sirolimus group and 5±3 days in the control group. The frequencies of abnormal cycles were higher among rats receiving sirolimus: 50% in the sirolimus group and 16% in the control group. Sirolimus blocked the mTOR pathway and amplified signaling in ovarian follicles through the pro-proliferative phosphatidylinositol 3-kinase pathway (**Figure 4**).

Serum levels of follicle stimulating and luteinizing hormones (FSH, LH) were similar between groups: FSH: 1.8±0.3 and 2.2±0.3 nanograms per milliliter in the sirolimus and control groups respectively; LH: 3.4±0.7 and 3.1±0.4 nanograms per milliliter in the sirolimus and control groups respectively.

## Discussion

When given sirolimus at a relatively low dose for a median of 19 months, 52% of 21 patients with ADPKD reported menstrual cycle abnormalities, compared to 17% of 18 patients in the control group. Sirolimus also increased the risk of ovarian cysts, and one patient was cystectomized after receiving sirolimus for five months. With relatively few female patients, it is not possible to make precise estimates but these increases in relative risk are potentially large – with a fourfold increase or more possible.

Reports from other studies suggest a lower ovarian toxicity or make no mention of it at all. In a multi-center placebo controlled phase II trial, Kahan and colleagues did not report menstrual cycle abnormalities in 47 female kidney allograft recipients receiving sirolimus for 12 months [4]. In two multi-center placebo controlled phase III trials, 180 and 149 female kidney allograft recipients were given sirolimus for 12 and 6 months respectively and there were no reports of menstrual cycle disturbance or ovarian cysts among these patients [2], [3]. This discrepancy is likely explained by the systematic and prospective assessment for menstrual cycle and ovarian cyst formation in our study which was not done in these large multicenter studies.

Our findings of sirolimus associated ovarian toxicity are supported by two reports of a higher frequency of oligoamenorrhea and ovarian cysts in pancreatic islet recipients treated with a dual therapy of sirolimus and tacrolimus [6], [7]. Cure and colleagues reported a higher frequency of oligoamenorrhea after transplantation and the occurrence of ovarian cysts in 8 of 13 patients, while serum follicle and luteinizing hormone levels remained in the normal range [6]. Alfadhli and colleagues reported on the occurrence of ovarian cysts in 31 of 44 premenopausal patients 8 months after transplantation and then after sirolimus withdrawal, a cyst size reduction in 80% of these 31 patients [7].

Sirolimus-associated adverse events of this sort are usually not assessed in randomized trials, even though normal menstrual cycle recovery after successful organ transplantation is a major benefit and a biological marker of general health, and menstrual cycle abnormalities are a sign of lower fertility and are associated with cardiovascular disease and bone loss.[32], [33] In clinical practice, sirolimus is often given simultaneously with other immunosuppressants making it difficult to reliably link observed ovarian toxicity with a single immunosuppressive agent.

In our phase II trial, we enrolled patients with early stage autosomal dominant polycystic kidney disease. The disease is characterized by the cystic enlargement of both kidneys while glomerular filtration rate remains preserved up to age 40 in most patients [34], [35]. There is no evidence that ADPKD affects the menstrual cycle or fertility in woman with normal renal function [11], [36]. Cysts can also occur infrequently in organs other than the kidneys;[37] however, the frequency of ovarian cysts, ovarian size and follicle dimensions are no different in women with ADPKD than in a normal population [9], [10]. Our cyst measurements may have included normal pre-ovulatory follicles as false positives. However our analyses show that sirolimus increased the risk of both ovarian cysts and menstrual cycle disturbances in patients receiving sirolimus and these differences between randomized groups suggest that the cysts detected in patients treated with sirolimus were probably not normal.

The sirolimus target mTOR is a key regulatory kinase of cell growth, proliferation and differentiation and is also expressed in non-T-cells, which can lead to unexpected toxicities including the ovaries. Animal studies indicate that the mTOR signaling pathway regulates luteal hormone receptor action [38], the follicle-stimulating hormone signaling pathway [39], granulosa [40] and luteal [41] cell proliferation and control of primordial oocyte dormancy [42]. Shivaswamy and colleagues reported an increased frequency of abnormal cycles and hyperinsulinemia in Sprague-Dawley rats receiving daily 2 milligrams sirolimus subcutaneously for 4 weeks, but a similar frequency of abnormal ovarian follicles in the their sirolimus and control groups [19]. We also observed an increased frequency of abnormal cycles in Wistar rats receiving sirolimus for 3 weeks, and a similar number and morphology of ovarian follicles in our sirolimus and control groups.

Polycystic ovary syndrome (PCOS) manifests as menstrual cycle disturbances, ovarian cysts, and insulin resistance [43]. Sirolimus causes insulin resistance in rodents [19] and in humans [44], [45] and we observed cycle disturbance and ovarian cysts in our ADPKD patients when treated with sirolimus, suggesting sirolimus promotes a polycystic ovary like syndrome. In our animal study, sirolimus activated Akt in ovaries. Polymorphisms of the Akt gene were associated with an increased risk for the occurrence of PCOS in epidemiological studies [46] and an increase in the insulin-induced IRS/PI 3-kinase/Akt pathway in rat ovaries caused a PCOS like phenotype [47], suggesting this pathway has a role in the development of ovarian cysts.

Our results should be interpreted in the context of the trial setting. Even though the achieved sirolimus dose was approximately 35% lower than the intended dose, mainly because of dose-limiting gastrointestinal side effects, our findings show evidence of sirolimus-associated ovarian toxicity. Close monitoring is therefore prudent for patients receiving sirolimus and this might help guide clinical use of mTOR inhibitors.

## Supporting Information

Checklist S1 **CONSORT Checklist.**
(PDF)

Protocol S1 **Trial Protocol.**
(PDF)
