# Supplementary material for: Low-Dose Oral Sirolimus and the Risk of Menstrual-Cycle Disturbances and Ovarian Cysts: Analysis of the Randomized Controlled SUISSE ADPKD Trial
Source: PLoS One. 2012 Oct 10;7(10):e45868. doi: 10.1371/journal.pone.0045868 (PMC3468602; doi:10.1371/journal.pone.0045868)
Supplement: Protocol S1 — Trial Protocol. (PDF) [file pone.0045868.s002.pdf]

Clinical proof-of-concept trial to assess the therapeutic effect of sirolimus in patients with autosomal dominant polycystic kidney disease: SUISSE ADPKD study

Andreas L. Serra[1§], Andreas D. Kistler[1], Diane Poster[1], Marian. Struker[1], Rudolf P. Wüthrich[1], Dominik Weishaupt[2] and Frank Tschirch[2]

[1]Clinic for Nephrology, University Hospital, CH-8091 Zürich, Switzerland
[2]Institute of Diagnostic Radiology, University Hospital, CH-8091 Zürich, Switzerland

[§]Corresponding author

Email addresses:

ALS	andreas.serra@usz.ch
ADK	andreas.kistler@usz.ch
DP	diane.poster@usz.ch
MS	marian.struker@usz.ch
RPW	rudolf.wuethrich@usz.ch
DW	dominik.weishaupt@usz.ch
FT	frank.tschirch@usz.ch

Version 27.06.2007
Work count: 2631

Article published as:
Serra AL, Kistler AD, Poster D, et al. Clinical proof-of-concept trial to assess the therapeutic effect of sirolimus in patients with autosomal dominant polycystic kidney disease: SUISSE ADPKD study. BMC Nephrol 2007;8:13.

Address correspondence to: Andreas L. Serra, M.D.
Clinic for Nephrology
University Hospital
Rämistrasse 100
8091 Zürich
Switzerland
Tel. +41 44 255 33 84
Fax. +41 44 255 45 93
E-mail andreas.serra@usz.ch

Abstract

Background

Currently there is no effective treatment available to retard cyst growth and to prevent the progression to end-stage renal failure in patients with autosomal dominant polycystic kidney disease (ADPKD). Evidence has recently been obtained from animal experiments that activation of the mammalian target of rapamycin (mTOR) signaling pathway plays a crucial role in cyst growth and renal volume expansion, and that the inhibition of mTOR with rapamycin (sirolimus) markedly slows cyst development and renal functional deterioration. Based on these promising results in animals we have designed and initiated the first randomized controlled trial (RCT) to examine the effectiveness, safety and tolerability of sirolimus to retard disease progression in ADPKD.

Method/design:

This single center, randomised controlled, open label trial assesses the therapeutic effect, safety and tolerability of the mTOR inhibitor sirolimus (Rapamune®) in patients with autosomal dominant polycystic kidney disease and preserved renal function. The primary outcome will be the inhibition of kidney volume growth measured by magnetic resonance imaging (MRI) volumetry. Secondary outcome parameters will be preservation of renal function, safety and tolerability of sirolimus.

Discussion:

The results from this proof-of-concept RCT will for the first time show whether treatment with sirolimus effectively retards cyst growth in patients with ADPKD.

Trial registration: NCT00346918

Background

Autosomal dominant polycystic kidney disease (ADPKD) is the most common hereditary cause of end-stage renal disease (ESRD), affecting all ethnic groups worldwide, with an incidence of 1 in 500 to 1 in 1000 [1]. ADPKD is characterized by the progressive development of innumerable cysts in both kidneys, which distort the normal kidney architecture and leads to a loss of renal function. The development of renal failure is highly variable, but typically patients develop ESRD by the age of 40 to 50 years, necessitating renal replacement therapy (RRT) and/or kidney transplantation [2]. Apart from blood pressure control and symptomatic treatment of cyst bleedings and infections there is no curative therapy for this disease [3]. PKD1 and PKD2 encode the proteins polycystin-1 and polycystin-2 which are expressed in the kidney and function together to regulate growth and morphologic configuration of renal epithelial cells [4]. Mutation in PKD1 leads to a more severe phenotype of ADPKD than mutations in PKD2, with ESRD occurring on average 20 years earlier (53.4 versus 72.7 years) [5].

In ADPKD progressive cyst growth generally precedes the development of renal insufficiency. Compensatory mechanisms (hyperfiltration) maintain renal function virtually normal for decades despite continuous cyst growth. By the time renal function starts to decline, the kidneys are usually grossly enlarged with little normal renal parenchyma recognisable on imaging studies. Data from the consortium for radiologic imaging studies of polycystic kidney disease (CRISP) and others have shown that the rate of kidney volume growth is a predictor of renal functional decline and therefore kidney volume is used as surrogate marker of disease progression especially in clinical intervention trials for ADPKD [6, 7]

Non-invasive radiologic methods are available to monitor the growth rate of kidney volume. Renal ultrasound measurements are operator-dependent and not precisely reproducible. Unenhanced and contrast enhanced Computer tomography (CT) scanning is reported to be an accurate method to determine kidney volume, but it involves ionizing radiation and potentially nephrotoxic contrast medium and is therefore not an ideal method in patients with reduced kidney function needing repetitive measurements [8, 9]. Due to its high soft tissue contrast and the lack of ionizing irradiation Magnetic resonance imaging (MRI) is also considered useful to monitor kidney volume changes in ADPKD. The analysis of sequential

MRI scans was shown to be accurate to monitor rates of kidney volume enlargement in ADPKD [7].

Sirolimus is an immunosuppressant that binds to FK Binding Protein-12 (FKBP-12) and inhibits the activation of the mTOR, a key regulatory kinase of growth and proliferation. Sirolimus is approved for the prevention of graft rejection following renal transplantation. Due to its antiproliferative properties it is also used in coated stents to prevent coronary artery restenosis after angioplasty [10]. Furthermore it has shown clinical effectiveness in kidney transplant recipients with Kaposi's sarcoma [11].

We have shown previously that the mTOR inhibitors rapamycin and everolimus effectively reduce cyst growth and loss of renal function in an experimental animal model for PKD [12, 13]. Additional studies have shown that rapamycin is also effective in various mouse models of polycystic kidney disease, including dominant and recessive forms [14]. Of interest, an analysis of ADPKD patients which received a renal transplant, revealed that cystic kidney volumes regressed under immunosuppression with sirolimus [15]. Based on these promising results we have designed and initiated the first clinical trial to examine the effectiveness and safety of sirolimus in young patients with early manifestations of ADPKD and intact renal function.

Methods/Design

Study aim

The primary objective of the SUISSE ADPKD study is to assess the effectiveness of sirolimus to retard kidney volume growth and to prevent the loss of renal function in young patients with ADPKD and preserved renal function. Patients with ADPKD and kidney volume growth that can be documented within 6 months are randomized to treatment with sirolimus 2 mg/day for 18 months (Figure 1) or standard treatment. The secondary objectives are to follow renal function and blood pressure and to monitor for the occurrence of proteinuria. Safety and tolerability of sirolimus treatment in ADPKD patients will also be assessed.

Study design and setting

The study is a single center randomised controlled open label trial which is open to ADPKD patients with a positive family history of ESRD due to ADPKD thereby selecting mostly

patients with mutations in the PKD1 gene. The study will involve 100 ADPKD-patients aged 18-40 years with a creatinine clearance >70 ml/min. Kidney volumes will be measured by MRI without contrast media at study month 0 and 6. Patients with documented volume progression are randomized at a 1:1 ratio to sirolimus 2 mg/day or standard treatment for 18 months. Recruitment has started in May 2006 and will last until December 2007. The study will be completed by December 2009.

Ethical considerations

Ethical approval has been obtained form the local ethics committee of the University Hospital Zürich.

Study drug and dosing

Rapamune® (sirolimus) will be used at a fixed dose of 2 mg daily to achieve sirolimus through levels between 4-10 µg/L. By using this rather low dose of sirolimus as monotherapy we expect a low incidence of sirolimus-related adverse events. The dose will be reduced or withheld in case of a through level exceeding 10 µg/l, elevated liver enzymes (> 2-fold above normal values), thrombopenia (< 100'000/mm^3), leukopenia (< 3'000/mm^3) or serious sirolimus-associated toxicity.

Study drug adherence

Adherence to the prescribed study drug will be assessed by using the Medication Event Monitoring V Track-Cap System (MEMS®, Aardex, Ltd., Zug, Switzerland) during the complete treatment period. The MEMS® assesses the medication adherence reliably and sensitive as a period with a lack of medication bottle opening documentation, representing most likely an episode of non-adherence [16, 17]. The system monitors electronically the date and time of the medication bottle opening. We will measure 1) treatment adherence as the proportion of medication vial caps opened in a given month relative to the prescribed doses for that month, 2) dosing adherence as the percentage of days with correct dosing and 3) drug holidays, as the number of periods without drug intake that exceeded 48 hours.

Identification of eligible patients

Study participation is offered to all eligible patients with ADPKD. ADPKD patients suffering from advanced renal failure including dialysis patients and transplant recipients treated at our clinic are informed about the study and screening for ADPKD is offered to their relatives. All nephrology clinics and dialysis units in Switzerland have been informed and information material (print and in the internet [18]) for study participants and health professionals has

been provided. Potential study participants are invited for a screening visit including medical history, physical examination, renal ultrasonography and blood and urine analyse.

Male or female ADPKD patients aged 18 to 40 years with a creatinine clearance ≥ 70 ml/min are eligible for the study if they exhibit a kidney volume progression over the observational pre-randomisation period of 6 months. The diagnosis of ADPKD is based on ultrasonographic diagnostic criteria in patients with a family history of polycystic kidney disease [19]. In patients with negative family history, proof of a mutation in the PKD1 or PKD 2 genes is required for inclusion (sequencing analysis; Athena Diagnostics, Inc., Worcester, MA, USA). Detailed study inclusion and exclusion criteria are given in table 1 and 2.

Randomization and study blinding

Patients are randomized at a one to one ratio to sirolimus or standard treatment alone. The randomisation list has been generated by a biostatistics unit which is independent of the study team using a permuted blocks design with a random block size of 4 or 6 to guarantee a balanced allocation. The randomisation codes are kept in sealed sequentially numbered opaque envelopes and are not opened until two MR scans within 6 months have shown an enlargement of the total kidney volume of ≥ 2 %.

Primary outcome

The primary objective of the SUISSE ADPKD study is to determine the effect of sirolimus treatment on kidney volume enlargement in ADPKD patients with preserved renal function. Patients with documented kidney volume growth in the last six months will be randomized to sirolimus or standard treatment. Kidney volume will be measured 6 and 18 months after randomization and the percent annual growth of the combined (left and right) kidney volume will be calculated.

Secondary outcome

Absolute kidney volume growth from inclusion to month 18 will be assessed as a secondary outcome. Other secondary objectives are to compare blood pressure, renal function and proteinuria in patients with sirolimus or standard treatment and to assess safety and tolerability of sirolimus treatment. New onset or progression of arterial hypertension might reflect disease progression of ADPKD or a potential adverse drug effect and will be assessed comparing the change of blood pressure and the change of antihypertensive drug dosage during follow up. Renal function will be assessed by using estimation equations (Cockcroft-Gault and cystatin C) as well as measured creatinine clearance determined by 24-hour urine

collection. Proteinuria will also be measured by 24-hour urine collection. The frequency and severity of all reported adverse events will be recorded, including laboratory abnormalities such as anemia, thrombocytopenia and hyperlipidemia.

Magnetic resonance imaging

All individuals undergo MR imaging of the kidneys using a 1.5 Tesla scanner. For signal reception in all examinations an 8-channel anteroposterior phased-array surface coil (torso array coil) is placed around the patient and covers the entire kidneys. The imaging protocol includes unenhanced sequences only. In order to get an overview of the extent of the cystic disease of the kidneys a coronal single shot fast spin echo (SSFSE) sequence is acquired in breath hold technique. The MR imaging parameters of this sequence are as follows: repetition time (TR) msec/echo time (TE) msec 1349/90.1; field of view 48x48 cm; acquisition matrix 384x224; section thickness 4 mm; no interslice gap. Based on this coronal sequence the transaxial sequences are planned. The transaxial sequences consists of two breath hold T1-weighted fast spoiled gradient echo (FSPGR) sequences (TR msec/TE msec= 85/1.4) with two different slice thicknesses (3 and 4 mm, respectively). Other parameters of the T1-weighted gradient-echo pulse (GRE) sequences are: field of view 48x48 cm, matrix 256x160; no interslice gap. In addition a transaxial T2-weighted fast spin echo (FSE) sequence with respiratory triggering is performed (TR msec /TE msec = 17143/102.8; field of view 48x48 cm, matrix 256x160, thickness 3 mm; no interslice gap). To measure the kidney volumes the transaxial breath hold T1-weighted FSPGR sequence with a slice thickness of 3 mm is primarily used. In case of a more advanced disease with large polycystic kidneys and/or when respiratory artefacts are present the volume measurements are either performed on the transaxial breath hold T1-weighted FSPGR sequence with a slice thickness of 4 mm or on the transaxial T2-weighted FSE sequence. Once one of these sequences is chosen the same sequence is used for volume measurements in the following MR imaging sessions.

Renal volume measurements

Two independent trained observers perform a manual segmentation of both kidneys for each patient. To prevent bias, the observers are blinded to all clinical and radiological data, their first measurements and the results of the other observer. The measurements are performed in random order. Blinding is performed with regard to patients and the different time points when imaging has been obtained. Manual segmentation is performed electronically on an interactive workstation (Advantage Windows Workstation; GE Medical Systems Europe, Buc, France). Each kidney is assessed separately. On each section, the outlines of the kidney are manually drawn by using the computer mouse. The vessels and the ureter in the area of

the renal hilum are excluded from manual volumetric marking. The volume corresponding to each outline is obtained by multiplying the area of the outline by the section thickness. The total volume of the kidney segments is obtained by summing the volume of each section. The manufacturer's software automatically calculates the total volume after drawing the outlines of the kidney on all sections. Mean values of the measurements performed by the two independent observers will be used for analysis. In case of a large disagreement, both observers will repeat measurement.

Data collection

Data will be collected into a web-based data base designed to capture all visit information including medical history, results form laboratory analysis and adverse events. Baseline data and ongoing data collection as outlined in table 3 will be obtained.

Patient follow-up procedures

Study duration for all patients will be 24 months in total, consisting of a 6 months pre-randomisation observational period and 18 months follow up after randomisation (Figure 1). Four main study visits including kidney MRI and 24h urine collection will take place at baseline, month 6, 12 and 24. Randomisation and inclusion in the study takes place after evaluation of kidney volume growth from baseline to month 6 within 2 weeks after the second MRI. Three additional visits including clinical assessment and blood and urine chemistry will take place at month 9, 15 and 18. Patients in the treatment arm will have four extra visits at week 2, 4, month 1 and 2 after randomisation to allow for blood level monitoring and potential dose adjustment of the study medication. Patients without volume progression during the pre-randomisation period will be followed by MRI for additional 6 months and enrolled if kidney volume enlargement is detectable.

Study withdrawal

Patients will be censored and withdrawn from follow-up at their request. Patients will also be censored if they are not randomized after the pre-randomization observation period.

Statistical analysis

Principal analysis will be undertaken using an intention-to-treat approach. A secondary on-treatment analysis will also be performed. The annual percent change in kidney volume will be determined by regressing the log transformed total kidney volume at month 6, 12 and month 24 against time for each patient through the least squares method. Mean annual decline of renal function will be calculated by regressing GFR or creatinine clearance against time. All primary and secondary end point variables will be compared using a two-sided α-level of

0.05. To account for possible baseline imbalances, a secondary analysis will be performed in which comparison of treatment groups for all endpoints will be adjusted for predefined covariates using multiple regressions. The predefined covariates are age, sex, presence of hypertension, medication with angiotensin converting enzyme inhibitors or angiotensin receptor blockers, baseline total kidney volume and percentage kidney growth during the pre-randomisation period.

Sample size considerations

In a large cohort of ADPKD patients, the mean annual kidney volume growth rate was 5.27% ± 3.92% (SD)[7]. Because patients with lack of progression during the pre-randomisation period will be excluded from our study, we expect to select for a higher progression rate in our study population. Due to a shorter observation interval compared to the mentioned observational study, the standard deviation might be higher. Presuming an annual kidney growth rate of 6% ± 4.75% (SD) in the control group, a sample size of 40 patients per group will have 80% statistical power to detect a 50% relative reduction of kidney volume growth using a two-sided α-level of 0.05. To account for a drop out rate of up to 20%, we plan to randomise a total of 100 patients.

Discussion

The SUISSE ADPKD study seeks to determine if sirolimus halts kidney volume growth in patients with ADPKD early in the disease course. Study participation is restricted to young patients with preserved kidney function because any effective treatment of ADPKD needs to be started early in the course of the disease to have an impact on long term kidney function. Our trial is the first clinical study addressing this question. If sirolimus treatment can reduce or stop volume growth in patients with maintained kidney function and prior documented kidney volume progression, these ADPKD patients could benefit the most from a treatment with an anti-proliferative agent like sirolimus. Similar studies using specific mTOR inhibitors to halt disease progression have been announced by others (Mayo Clinic, USA; Certican® trial, Novartis, Germany). We anticipate that sirolimus might be an effective therapeutic option for ADPKD patients that are prone to progress to end-stage renal disease.

Competing interests

The author(s) declare that they have no competing interests.

Authors' contributions

ALS, ADK and RPW were responsible for identifying the research question and drafting the study protocol. All authors have contributed to the development of the protocol and study design, as members of the study team. ALS and FT were responsible for drafting of this manuscript and all authors provided comments and have read and approved the final version.

Acknowledgements

The SUISSE ADPKD trial is an investigator-initiated clinical trial. We thank Wyeth Europe for financial support and for providing the study drug Rapamune®. Wyeth Europe had no role in the design or conduct of the study, or in the writing and submission of the manuscript. We thank M. Sollberger for secretarial assistance.

References

1. Dalgaard OZ: **Bilateral polycystic disease of the kidneys; a follow-up of two hundred and eighty-four patients and their families**. *Acta Med Scand Suppl* 1957, **328**:1-255.
2. Hateboer N, v Dijk MA, Bogdanova N, Coto E, Saggar-Malik AK, San Millan JL, Torra R, Breuning M, Ravine D: **Comparison of phenotypes of polycystic kidney disease types 1 and 2. European PKD1-PKD2 Study Group**. *Lancet* 1999, **353**(9147):103-107.
3. Wilson PD: **Polycystic kidney disease**. *N Engl J Med* 2004, **350**(2):151-164.
4. Harris PC, Rossetti S: **Molecular genetics of autosomal recessive polycystic kidney disease**. *Mol Genet Metab* 2004, **81**(2):75-85.
5. Torra R, Badenas C, Darnell A, Nicolau C, Volpini V, Revert L, Estivill X: **Linkage, clinical features, and prognosis of autosomal dominant polycystic kidney disease types 1 and 2**. *J Am Soc Nephrol* 1996, **7**(10):2142-2151.
6. Franz KA, Reubi FC: **Rate of functional deterioration in polycystic kidney disease**. *Kidney Int* 1983, **23**(3):526-529.
7. Grantham JJ, Torres VE, Chapman AB, Guay-Woodford LM, Bae KT, King BF, Jr., Wetzel LH, Baumgarten DA, Kenney PJ, Harris PC *et al*: **Volume progression in polycystic kidney disease**. *N Engl J Med* 2006, **354**(20):2122-2130.
8. Thomsen HS, Madsen JK, Thaysen JH, Damgaard-Petersen K: **Volume of polycystic kidneys during reduction of renal function**. *Urol Radiol* 1981, **3**(2):85-89.
9. Ruggenenti P, Remuzzi A, Ondei P, Fasolini G, Antiga L, Ene-Iordache B, Remuzzi G, Epstein FH: **Safety and efficacy of long-acting somatostatin treatment in autosomal-dominant polycystic kidney disease**. *Kidney Int* 2005, **68**(1):206-216.
10. Morice MC, Serruys PW, Sousa JE, Fajadet J, Ban Hayashi E, Perin M, Colombo A, Schuler G, Barragan P, Guagliumi G *et al*: **A randomized comparison of a sirolimus-eluting stent with a standard stent for coronary revascularization**. *N Engl J Med* 2002, **346**(23):1773-1780.
11. Stallone G, Schena A, Infante B, Di Paolo S, Loverre A, Maggio G, Ranieri E, Gesualdo L, Schena FP, Grandaliano G: **Sirolimus for Kaposi's sarcoma in renal-transplant recipients**. *N Engl J Med* 2005, **352**(13):1317-1323.
12. Wahl PR, Serra AL, Le Hir M, Molle KD, Hall MN, Wuthrich RP: **Inhibition of mTOR with sirolimus slows disease progression in Han:SPRD rats with autosomal dominant polycystic kidney disease (ADPKD)**. *Nephrol Dial Transplant* 2006, **21**(3):598-604.
13. Wu M, Wahl PR, Le Hir M, Waeckerle-Men Y, Wuthrich RP, Serra AL: **Everolimus retards cyst growth and preserves kidney function in a rodent model for polycystic kidney disease**. *Kidney & Blood Pressure Research (in press)* 2007.
14. Tao Y, Kim J, Schrier RW, Edelstein CL: **Rapamycin markedly slows disease progression in a rat model of polycystic kidney disease**. *J Am Soc Nephrol* 2005, **16**(1):46-51.
15. Shillingford JM, Murcia NS, Larson CH, Low SH, Hedgepeth R, Brown N, Flask CA, Novick AC, Goldfarb DA, Kramer-Zucker A *et al*: **From the Cover: The mTOR pathway is regulated by polycystin-1, and its inhibition reverses renal cystogenesis in polycystic kidney disease**. *Proc Natl Acad Sci U S A* 2006, **103**(14):5466-5471.
16. Farmer KC: **Methods for measuring and monitoring medication regimen adherence in clinical trials and clinical practice**. *Clin Ther* 1999, **21**(6):1074-1090; discussion 1073.

17. Osterberg L, Blaschke T: **Adherence to medication**. *N Engl J Med* 2005, **353**(5):487-497.
18. **SUISSE ADPKD Study** [www.ADPKD.ch]
19. Ravine D, Gibson RN, Walker RG, Sheffield LJ, Kincaid-Smith P, Danks DM: **Evaluation of ultrasonographic diagnostic criteria for autosomal dominant polycystic kidney disease 1**. *Lancet* 1994, **343**(8901):824-827.

Tables

Table 1
Study inclusion criteria

•	Age 18 to 40 years
•	GFR ≥ 70 ml/min (Cockcroft – Gault formula)
•	Diagnosis of ADPKD:
	o Positive family history for ADPKD
	▪ patients < 30 years: ≥ 2 cysts in either kidney
	▪ patients ≥ 30 years: ≥ 2 cysts in each kidney
	o Negative family history for ADPKD but sonographically cystic kidney disease: proof of a mutation in the PKD1 or PKD2 gene is required (Athena Diagnostics, Inc., Worcester, MA, USA)
•	Documented kidney volume enlargement (MRI volumetry)
•	Signed informed consent

Table 2
Study exclusion criteria

•	Female patient of childbearing potential who is unwilling to use effective means of contraception
•	Increased liver enzymes (2-fold above normal values)
•	Hypercholesterolemia (fasting cholesterol > 8 mmol/l) or hypertriglyceridaemia (> 5 mmol/l) not controlled by lipid lowering therapy
•	Granulocytopenia (white blood cell < 3,000/mm3) or thrombocytopenia (platelets < 100,000/mm3)
•	Infection with hepatitis B or C, HIV
•	History of malignancy
•	Mental illness that interferes with the patient ability to comply with the protocol
•	Drug or alcohol abuse within one year of baseline
•	Co-medication with strong inhibitor of CYP3A4 and or P-gp like voriconazole, ketoconazole, diltiazem, verapamil, erythromycin or with a strong CYP3A4 and or P-gp inductor like rifampicin
•	Known hypersensitivity to macrolides or Rapamune®
•	Patients who are unwilling or unable to give informed consent

Table 3

Baseline and ongoing data collection

**Baseline data and follow-up data at month 6, 12 and 24**
MRI kidney volumetry
Creatinine clearance and estimated GFR (Cockcroft-Gault)
Proteinuria (24-hour urine and spot urine)
Physical examination and vital signs
Laboratory tests
Haematology/Biochemistry
Lipid profile
Sirolimus trough level
Pregnancy test[1]
Serological testing for hepatitis B, C and human immunodeficiency virus (HIV)[1]
MEMS® check
Adverse events and concomitant therapy
**Follow-up data every 3 months**
Serum creatinine
Proteinuria (spot urine)
Physical examination and vital signs
Laboratory tests
Haematology/Biochemistry
Lipid profile
Sirolimus trough level
Adverse events and concomitant therapy

[1]Only at baseline and month 24

Figure

Figure 1

Flow chart of SUISSE ADPKD study

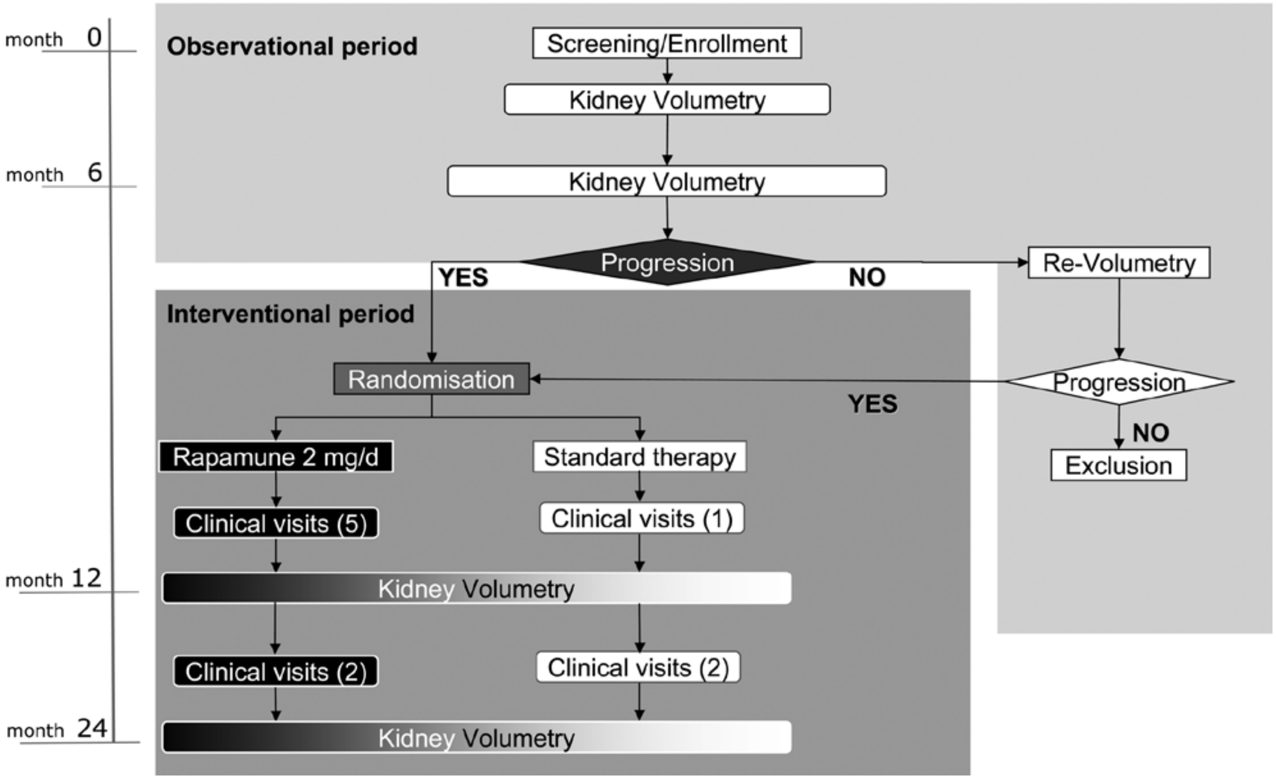

BIOP
Biometrical Practice

Sirolimus for patients with autosomal dominant polycystic kidney disease: a single center, randomized controlled open label trial

Author:	Jim Young, BIOP AG
Document type:	Statistical Analysis Plan
Development Phase:	Final Draft version 1.9
Release date:	03 April 2008

Table of contents

List of abbreviations and definition of terms....3
Statistical analysis plan....4
1 Objective....4
2 General....4
3 Populations....5
3.1 Intent-to-treat population....5
3.2 Per-protocol population....5
4 Interim analyses....5
5 Baseline and demographic characteristics....6
5.1 Derived variables....6
5.2 Primary efficacy evaluation....6
6 Secondary efficacy evaluation....7
7 Safety evaluation....8
8 Determination of sample size....8
9 Changes in the study conduct or planned analyses....9
9.1 Protocol amendments....9
9.2 Other changes in study conduct....9
10 References....9
11 Appendix I: Tables and figures....11
11 Appendix I: Tables and figures....11
11.1 Tables....11
11.2 Figures....16
12 Appendix II: Visit schedule....17
13 Appendix III: Laboratory parameters and vital signs....18

List of abbreviations and definition of terms

ICH	International Conference on Harmonisation
CONSORT	Consolidated Standards of Reporting Trials
CRF	case report form
ITT	intent to treat
ADPKD	autosomal dominant polycystic kidney disease
MRI	magnetic resonance imaging
MedDRA	Medical Dictionary for Regulatory Activities
WHO	World Health Organization
AE	adverse event

Statistical analysis plan

1 Objective

In this single center open label trial, patients with autosomal dominant polycystic kidney disease (ADPKD) are randomized to either sirolimus in addition to standard therapy or standard therapy alone. Those randomized to sirolimus have extra study visits during the first two months after randomization for potential dose adjustment. Those randomized to standard therapy are likely to receive only medication for blood pressure control and symptomatic treatment for cyst bleeding because there is no known curative therapy for this disease. The trial has two phases: a screening phase of 6 months prior to randomization and a treatment phase after randomization of 18 months.

This analysis plan reflects both published and unpublished trial protocols [1,2]. Attention will be drawn to any recommendation in this plan that implies a major amendment or addition to these protocols. The analysis plan is designed to provide analytic results both suitable for publication in a high quality scientific journal and consistent with IHC guideline E9 ‘Statistical principles for clinical trials’ [3] and the CONSORT Statement [4].

2 General

For the purposes of this plan, baseline is when randomization takes place and this will be denoted as Day 1, so that screening begins at –6 months, and the treatment phase of the trial ends at 18 months. Note that during the treatment phase, there are visits at 3, 6, 9, 12 and 18 months for patients in both randomized groups and four additional visits for patients in the treatment group (at 2 and 4 weeks and at 1 and 2 months). These additional visits allow for dose adjustment.

The data will be entered, stored and analyzed by a contract research organization (Biometrical Practice BIOP AG, Basel), using version 8.2 of the SAS statistical package.

Information collected on the CRF was summarized according to the type of variable. For continuous variables the following summary statistics will be calculated:

Mean
Standard deviation
Median
Interquartile range
Range

For discrete variables the following summary statistics will be calculated:

Number of values in each category
Percentage in each category

All hypotheses tests and confidence intervals will be two sided and use a Type I error rate of $\alpha = 5\%$.

3 Populations

The principle efficacy analysis will use both the intent-to-treat (ITT) and per-protocol populations. The secondary efficacy analysis will use only the ITT. The safety analysis will use the safety population.

3.1 Intent-to-treat population

The intent-to-treat population is all randomized patients.

3.2 Per-protocol population

The per-protocol population is defined as patients included in the ITT population who did not have any major protocol violations. Major protocol violations are defined below.

3.2.1 Definition of protocol violations

Patients can withdraw from the trial at any time at their request. Protocol violations are defined as follows:

- Any patient in the ITT population not assessed at 18 months;
- Non-compliance to study treatment at 18 months – that is, either
  Less than 50% of study medication taken,
  OR
  More than 6 months continuously with no study medication.

3.2.2 Safety population

The safety population is all randomized patients.

4 Interim analyses

Interim statistical analyses have implications for the type I error rate. Without suitable adjustments to statistical methods, interim analyses can make it more difficult to publish a final analysis in a high quality journal (see [3] sections 4.1 and 4.5, also [5]).

Due to potential safety issues one interim analysis will be performed after 50 patients have completed the treatment phase. For the most part, the interim analysis will be a safety evaluation and will consider adverse events, but primary efficacy will also be evaluated. In order to protect the overall type I error rate of 0.05 (see [3] section 4.5), the O'Brien and Fleming boundary approach will be applied when testing the difference between randomized groups in the primary efficacy analysis (see [6]). This means that the standardized estimate of the difference between randomized treatment groups must be greater than 2.7967 in the interim analysis or greater than 1.9776 in the final analysis; these values correspond to nominal p-values of 0.005 and 0.048 in the interim and final analyses respectively.

5 Baseline and demographic characteristics

The inclusion and exclusion criteria are summarized in [2] in Tables 1 and 2. Important information includes: creatinine clearance (ml/min), how the diagnosis of ADPKD was made (family history and results of imaging), and kidney volume at screening and at randomization and percentage enlargement in kidney volume over this period.

Baseline data collection is summarized in [2] in Table 3. Important information includes: the results of a physical examination, the results of blood tests and of urine tests, and a summary of treatment history prior to screening and between screening and randomization.

Inclusion and exclusion criteria and baseline data will be summarized in standard BIOP tables – see Appendix.

5.1 Derived variables

Kidney volume will be assessed from magnetic resonance imaging (MRI). Each image will be assessed by two independent observers, blind to all clinical data and to the results of the other observer. Software will automatically calculate total kidney volume (left and right combined) after the outline of each kidney is drawn on all sections. The mean of the two assessments will be used for analysis.

5.2 Primary efficacy evaluation

The ITT and per-protocol populations will be used to evaluate primary efficacy.

Using change over time as an outcome does not correct for any baseline imbalance: 'if the treatment is at an unfair disadvantage compared to placebo when its effects are measured in raw outcomes (due to an imbalance in baselines), it will have an unfair advantage if change scores are used' ([7] section 7.2.3). Using an analysis of covariance with the baseline measure as a covariate (i.e. kidney volume at randomization) will give an appropriate adjustment for any baseline imbalance and an estimate of the difference between randomized groups that has a lower variance; that is, the analysis will have more power [8].

Assuming a log transformation is desirable so that the distribution of total kidney volume after 18 months treatment is approximately normal, the following analysis of covariance model is suggested for the primary efficacy analysis (see [7] section 7.2.6):

log(total kidney volume after 18 months treatment) =

$\beta_0 + \beta_1$.log(total kidney volume at baseline) + β_2.(randomized group indicator).

Because this is a more powerful analysis than the analysis originally proposed [2], the sample size in [2] will still be sufficient.

With this analysis, the estimate of the difference between randomized groups (the estimate of β_2) has a simple interpretation. A confidence interval for a difference on the log scale can be back-transformed to become a confidence interval for a ratio on the original scale. If the distribution of log transformed kidney volume is approximately normal, then a back-transformed confidence interval shows the multiplicative effect of treatment on the median

kidney volume. [This is nicely explained at http://www.tufts.edu/~gdallal/ci_logs.htm.] Such an analysis of covariance would provide the following information:

Primary efficacy outcome: log final total kidney volume (ie at 18 months)

Parameter	Meaning	Estimate	95% CI	p-value
$\exp(\beta_0)$	median final kidney volume			
$\exp(\beta_1)$	multiplicative effect of baseline kidney volume on median final kidney volume			
$\exp(\beta_2)$	multiplicative effect of treatment on median final kidney volume			

Such an analysis would not preclude presenting data in graphs showing change or percentage change over time.

A difficulty in any analysis is how to treat missing values when using the ITT population. The common practice of 'last observation carried forward' is probably not appropriate here. This is because progression over time is expected and an effective treatment will delay progression; therefore using a previous observation when patients drop out has the potential to make treatment look more effective than it really is. A conservative treatment of missing values is needed, and this will involve replacing missing values in the sirolimus group by the upper quartile increase in kidney volume seen in this group and replacing missing values in the standard therapy group by the lower quartile increase in kidney volume seen in this group.

6 Secondary efficacy evaluation

All secondary efficacy analyses will use the ITT population. It is important to specify all secondary analyses in this analysis plan. Any unplanned analyses must be acknowledged when publishing and these could make it more difficult to publish the final analysis in a high quality journal (see [3] sections 5.1 and 7.1, also [9]).

A second efficacy analysis of total kidney volume will include additional covariates. As a general rule, 10-15 patients are needed per model predictor; otherwise estimates are not reliable (see [10]). Therefore at most four additional covariates should be added to the analysis of covariance model previously described, giving six model predictors in total. The following additional covariates are to be added to the model for kidney volume at 18 months:

(1) age;

(2) sex;

(3) total kidney volume at screening;

(4) protein creatinine ratio from the spot urine at screening.

For other secondary efficacy outcomes, analysis will be by analysis of covariance with the outcome at baseline as the only covariate and where necessary, using an appropriate

transformation (log or logit). Missing outcomes will be replaced by carrying the last observation forward. If any emphasis is placed on a specific secondary efficacy outcome, it would be prudent to consider whether this replacement policy is conservative for that particular outcome. In general, however, the overall pattern across secondary efficacy outcomes will be of more interest than any one particular outcome.

Secondary efficacy outcomes include:

(1) left and right kidney volume (LKV, RKV);

(2) total cyst volume;

(3) renal function (as assessed by estimated creatinine clearance according to the Cockcroft Gault formula, and by the albumin creatinine ratio in spot-urine);

(4) blood pressure (both diastolic and systolic);

(5) any use of anti-hypertensive medication (ACEI, ARB, BB, diuretics, others);

(6) total liver volume (TLV).

7 Safety evaluation

The safety population will be used to evaluate safety. In a publication, only a summary of the most important information will be reported. However detailed records must be available.

The frequency and severity of all reported adverse events should be summarized in tables, including vital signs and laboratory abnormalities (see Appendix). Adverse events will be coded by assigning the preferred term from the MedDRA dictionary. Adverse events will be summarized by presenting the number and percentage of patients having any adverse event, having an adverse event in each system organ class and having an adverse event with a particular preferred term within a system organ class. Summaries for each randomized group will be given for the whole study (screening and treatment phases) and for the period from 3 to 18 months (post-dose adjustment).

Severe adverse events, events that results in a patient discontinuing and events that cause a dose reduction in sirolimus after randomization should be itemized in tables and noted in a publication. Patients who elect to withdraw from the study or are withdrawn by an investigator should be itemized in a table together with the reasons for withdrawing.

Adherence to treatment will be documented by: the proportion of medication vial caps opened in a given month relative to the prescribed doses for that month (‘treatment adherence’); the percentage of days with the correct dosing (‘closing adherence’); and the number of periods exceeding 4 days without drug intake (‘drug holidays’).

8 Determination of sample size

The sample size of 40 patients per randomized group is based on an expected annual kidney volume growth rate of 6% (SD 4.75%) in the control group and an 80% probability of detecting a 50% relative reduction in this growth rate under treatment. A drop-out rate of 20% is expected, so that 100 patients will be randomized, but patients who drop-out will be included in the analysis and not replaced.

9 Changes in the study conduct or planned analyses

9.1 Protocol amendments

This analysis plan amends the primary efficacy analysis in the protocol and adds a single interim analysis of primary efficacy.

9.2 Other changes in study conduct

None planned.

10 References

1 Serra, A. L., Wüthrich, R. P, and Kistler, A. D. Sirolimus (Rapamune) für Patienten mit autosomal dominanter polyzystischer Nierenerkrankung (ADPKD): eine randomisierte, kontrollierte Studie [Studienprotokoll]. Klinik für Nephrologie, Department für Innere Medizin, Universitätsspital Zurich; 2006.

2 Serra AL, Kistler AD, Poster D et al. Clinical proof-of-concept trial to assess the theraputic effect of sirolimus in patients with autosomal dominant polycystic kidney disease: SUISSE ADPKD study. BMC Nephrology 2007;8:13, http://www.biomedcentral.com/1471-2369/8/13.

3 ICH Harmonised Tripartite Guideline. Statistical principles for clinical trials. International Conference on Harmonisation E9 Expert Working Group. Statistics in Medicine 1999;18:1905-42.

4 Moher D, Schulz KF, Altman DG. The CONSORT statement: revised recommendations for improving the quality of reports of parallel-group randomised trials. Lancet 2001;357:1191-4.

5 Schulz KF, Grimes DA. Multiplicity in randomised trials II: subgroup and interim analyses. Lancet 2005;365:1657-61.

6 Wang SK, Tsiatis AA. Approximately optimal one-parameter boundaries for group sequential trials. Biometrics 1987;43:193-9.

7 Senn, S. Statistical issues in drug development. Chichester: Wiley; 1997.

8 Pocock SJ, Assmann SE, Enos LE, Kasten LE. Subgroup analysis, covariate adjustment and baseline comparisons in clinical trial reporting: current practice and problems. Stat Med 2002;21:2917-30.

9 Schulz KF, Grimes DA. Multiplicity in randomised trials I: endpoints and treatments. Lancet 2005;365:1591-5.

10 Babyak MA. What you see may not be what you get: a brief, nontechnical introduction to overfitting in regression-type models. Psychosom Med 2004;66:411-21.

11 Appendix I: Tables and figures

11.1 Tables

11.1.1 Patient disposition

Standard BIOP tables will be used to show patient flow (ie Tables 1 to 3).

Table 1. Patient disposition

	Sirolimus	Standard therapy	Total
**Total no. of patients** - n(%)			
Screening			
Kidney volumetry (Visit 1)			
Kidney volumetry (Visit 2)			
Randomization (Visit 3)			
3 months treatment (Visit 8)			
6 months treatment (Visit 9)			
9 months treatment (Visit 10)			
12 months treatment (Visit 11)			
18 months treatment (Visit 12)			
**Summary of patients**			
Screening			
Progression (first 6 months)			
Progression (second 6 months)			
Randomized			
Premature discontinuations			
Completed study treatment			

Table 2. Patient disposition: premature discontinuations (all randomized patients)

	Sirolimus	Standard therapy	Total
**Discontinuations** - n(%)			
Total			
Death			
Adverse events / illnesses			
Abnormal lab test			
Lack of efficacy			
Protocol violations			
Administrative / other			

Table 3. Major protocol violations (all randomized patients)

	Sirolimus	Standard therapy	Total
**Major protocol violations**			
Total (at least one PV)			
Major PV 1			
Major PV 2			
Major PV 3			

11.1.2 Randomized group comparisons

Standard BIOP tables will be needed to compare randomized groups (ie Table 4). Tables will display the following information:

- demographics at randomization;
- hepatitis and HIV serology by visit (second kidney volumetry and 18 months);
- stimulant use (nicotine, alcohol, coffee) at first kidney volumetry;
- MRI volumetry by visit (first kidney volumetry, second kidney volumetry, 6, 12 and 18 months);
- ADPKD symptoms by visit (at all visits except screening);
- vital signs by visit (screening, first kidney volumetry, second kidney volumetry, randomization, 3, 6, 9, 12 and 18 months);

- blood chemistry by visit (screening, first kidney volumetry, second kidney volumetry, randomization, 3, 6, 9, 12 and 18 months);
- blood biochemistry by visit (screening, first kidney volumetry, second kidney volumetry, randomization, 3, 6, 9, 12 and 18 months);
- spot urine chemistry by visit (first kidney volumetry, second kidney volumetry, randomization, 3, 6, 9, 12 and 18 months);
- 24 hour urine chemistry by visit (first kidney volumetry, second kidney volumetry, 6, 12 and 18 months);
- concomitant medications by visit (all visits except screening).

Table 4. Demographic data (intent-to-treat population)

	Sirolimus	Standard therapy	Total
Age - (years)			
	.	.	.
N (%)			
Mean	.	.	.
S.D.	.	.	.
Median	.	.	.
Q1	.	.	.
Q3			
Range			
Sex – n(%)			
Male	.	.	.
Female	.	.	.
BMI - (kg/m^2)			
N (%)	.	.	.
Mean	.	.	.
S.D.	.	.	.
Median	.	.	.
Q1	.	.	.
Q3			
Range	.	.	.

11.1.3 Longitudinal comparisons

Tables showing data over time will be needed for primary and secondary outcomes:

- total kidney volume at first and second kidney volumetry, 6, 12 and 18 months;
- blood pressure at screening, first and second kidney volumetry, randomization, 3, 6, 9, 12 and 18 months;
- use of antihypertensive medication at randomization, 3, 6, 9, 12 and 18 months;
- proteinuria (albumin creatinine ratio and protein creatinine ratio) at screening, first and second kidney volumetry, randomization, 3, 6, 9, 12 and 18 months;
- renal function (estimated creatinine clearance according to the Cockcroft Gault formula, creatinine clearance based on results of 24 hour urine) at first and second kidney volumetry, 6, 12 and 18 months;
- vital signs (diastolic and systolic blood pressure, pulse, BMI), lipid (total, HDL and LDL cholesterol and triglycerides) and laboratory parameters (haemoglobin).

11.1.4 Sirolimus specific data

Tables showing data over time only in the sirolimus group will be needed for:

- blood pressure during the dose adjustment visits at two and four weeks and one and two months;
- use of antihypertensive medication during the dose adjustment visits at two and four weeks and one and two months;
- sirolimus dose at all post-randomization visits;
- sirolimus adherence at all post-randomization visits;
- sirolimus trough blood level at all post-randomization visits.

11.1.5 Adverse events

Serious adverse events will be listed and discussed. Any deaths that occur will be individually discussed.

Standard BIOP tables will be needed to summarize adverse events (Tables 5 and 6). Summaries will be given for the whole study (screening and treatment phases) and for the period from 3 to 18 months (post-dose adjustment). The following summaries are needed:

- incidence and severity of AE and SAE;
- incidence of AE leading to discontinuation from the study;
- incidence of AE leading to sirolimus dose reduction;
- incidence and severity of infections;
- incidence of each preferred term: anemia, neutropenia, leukopenia, thrompopenia;

- incidence and volume change of ovarian cysts;
- relative frequency and worst values of increased lipids and abnormal vital signs and laboratory parameters.

Abnormal thresholds are given in Appendix III for vital signs, lipid and laboratory parameters of interest. Note that lipid parameters are to be measured in the fasting state. High levels of blood glucose should be flagged as indicating either (pre-) diabetes or measurement not in the fasting state; the latter would have implications for other lipid parameters (such as triglycerides).

Table 5. Number of patients reporting AEs considered to be related to study treatment by system organ class and preferred term (safety population)

	Sirolimus	Standard therapy	Total
**Patients studied - n (%)**			
Total no. of patients studied	.		
Total no. of related adverse events			
Total no. of patients with an AE related to the study treatment	.		
**System organ class - n (%)**			
SOC 1	.	.	.
AE 1			
AE 2			
SOC 2	.	.	.
AE 1			
AE 2			
SOC 3	.	.	.
AE 1			
AE 2			

Table 6. Number of patients with any serious adverse events or adverse event which led to the discontinuation of the patient (safety population)

	Sirolimus	Standard therapy	Total
**Patients studied – n (%)**			
Total no. of patients studied	.	.	.
**Serious or significant events – n (%)**			
Death	.	.	.
Serious AEs	.	.	.
Clinically significant AEs	.	.	.
Discontinuations due to SAEs	.	.	.
Discontinuations due to clin. sig. AEs	.	.	.
..	.	.	.

11.2 Figures

Plots of primary and secondary outcomes are needed for total kidney volume (cm, % change from second volumetry), and for estimated and measured creatinine clearance (according to the Cockcroft Gault formula and based on results of 24 hour urine respectively) and albumin creatinine ratio in spot-urine.

12 Appendix II: Visit schedule

Visits are not always referred to in a consistent fashion – the various formats are listed below:

Function of visit	Approximate time	Online database	Data from database
Screening / enrolment	- 6 months	S (screening)	0
First kidney volumetry	- 6 months	V1	1
Second kidney volumetry	0 months	V2	2
Randomization	Day 1	V3	3
2 weeks dose adjustment	2 weeks	V4	6
4 weeks dose adjustment	4 weeks	V5	7
1 month dose adjustment	1 month	V6	8
2 months dose adjustment	2 months	V7	9
3 months treatment	3 months	V8	10
6 months treatment	6 months	V9	11
9 months treatment	9 months	V10	12
12 months treatment	12 months	V11	22
18 months treatment	18 months	V12	32

13 Appendix III: Laboratory parameters and vital signs

Notable laboratory parameters

Parameter	Units	Limits
**Electrolytes**		
Sodium	mmol/l	>150
		<125
Potassium	mmol/l	>6
		<3
**Liver function**		
SGOT (AST	U/l	>135
SGPT (ALT)	U/l	>135
GGT	U/l	>138
**Renal function, metabolic variables**		
Creatinine	µmol/l	>30% increase from visit 3 (V3)
Urea		None
Glucose	mmol/l	>13.9
		<2.5
**Infection**		
CRP	mg/l	>50
**Lipids**		
Cholesterol	mmol/l	>9.1
HDL	mmol/l	<0.5
LDL	mmol/l	>10
Triglycerides	mmol/l	>8.5
**Hematology**		
Hemoglobin	g/dl	<7
Leucocytes	x103/µL	<2
		>16
Platelets	x103/µL	<50
		>700
**Hematology:differential**		
Neutrophils	x103/µL	<1
Monocytes	x103/µL	>5
Eosinophils	x103/µL	>5
Basophils	x103/µL	>5
Lymphocytes	x103/µL	<1
**Spoturine**		
Albumin creatinine ratio	mg/mmol	>30

Notable vital signs

Vital sign	Units	Limits
Pulse	beats/min	>150
		<40
Systolic BP	mm Hg	>200
		<75
Diastolic BP	mm Hg	>115
		<40
Weight	Kg	None
